# Effects of Fines Content on Durability of High-Strength Manufactured Sand Concrete

**DOI:** 10.3390/ma16020522

**Published:** 2023-01-05

**Authors:** Sunbo Zheng, Jiajian Chen, Wenxue Wang

**Affiliations:** 1 Department of Civil Engineering, Foshan University, Foshan 528000, China; 2 Research Institute for Applied Mechanics, Kyushu University, Kasuga-Koen 6-1, Fukuoka 816-8580, Japan

**Keywords:** durability, fines content, granite, limestone, manufactured sand

## Abstract

Manufactured sand is one of the effective ways to alleviate the extreme shortage of natural sand in the construction industry. This paper uses granite and limestone manufactured sand to study the effect of high fines content on the durability of high-strength manufactured sand concrete, and analyzes its influence mechanism by combining macro and micro test methods. The results show that the carbonation depth of manufactured sand concrete is the smallest when the fines content is 10%. When the fines content is less than 15%, the chloride and sulfate impermeability of concrete are improved effectively. Through macroscopic and microscopic tests, it is found that the main reason why fines can improve the durability of concrete is the filling effect. Too much fines will inhibit the hydration of cement and adversely affect the durability of concrete. Therefore, the fines content of high-strength manufactured sand concrete should be controlled within 5~15%, and the durability is the best when the fines content is 10%.

## 1. Introduction

At present, sustainable development, energy conservation and CO_2_ emission reduction have become the focus of attention. They are the results of a series of ecological and environmental problems caused by excessive exploitation of non-renewable resources [1]. River sand is one of the important raw materials for producing concrete. As a developing country, the rapid development of urbanization in China has increased the demand for sand, leading to the depletion of high-quality river sand resources for many areas [2]. River sand is a non-renewable resource, and problems such as water and soil strata loss and river bank slippage caused by over-exploitation of river sand are becoming more and more prominent [3,4,5]. Therefore, in order to meet the rising demand for construction sand, alternatives to river sand must be found.

Manufactured sand is produced by crushing rocks, screening, washing, and other processes [6]. The manufactured sand has the characteristics of irregular particle shape and polygonal shape, high fines content and needle flakes, and poor particle size distribution. It is these characteristics that limit the popular use of the manufactured sand in concrete [7,8,9]. The clay content of manufactured sand is represented by the methylene blue (MB) value. Too large a MB value will lead to a significant increase in the early cracking and shrinkage of concrete, which will have adverse effects on the strength and durability. Fines is a particle with the same chemical composition as the parent rock that is inevitably produced in the production process of manufactured sand, and its particle size is less than 75 μm [10]. According to the Chinese construction sand standard, it is stipulated that the manufactured sand can contain a certain amount of fines. The maximum content of stone powder in machined sand is limited to 10%. Due to the limitations of the current technology and equipment for producing manufactured sand, the content of fines in the manufactured sand is difficult to meet the standard. According to the survey statistics, more than 60% of the 24 manufactured sand samples in the manufactured sand quarry in Guangdong Province did not meet the standard, that is, the fines content exceeded 10% [11].

Most of the excess fines in manufactured sand is removed by washing with water, but this method of processing fines wastes water resources and increases production costs. Moreover, during the process of washing, not only fines but also particles of 0.15 mm, 0.3 mm, and 0.6 mm or even larger are removed, which is harmful to the packing of the manufactured sand [12]. The lack of effective utilization of excess fines will result in waste of resources. Therefore, it is necessary to deal with the problem of excessive fines content in manufactured sand. The research shows that among the many characteristics of manufactured sand including MB value, fines content, and needle flake content, the most obvious influence on the performance of manufactured sand concrete is the fines content [13]. A certain amount of fines can effectively improve the workability of manufactured sand concrete [14,15]. At the same time, an appropriate amount of fines can improve the bonding of aggregate and cement, improve the packing of concrete and the mechanical properties of concrete. As a result, the performance of concrete at this time is not worse than that of river sand concrete [16,17,18,19,20]. However, excessive fines content will negatively affect the performance of concrete [21,22].

The increase of fines content is conducive to the improvement of concrete strength, but with the further increase of fines content, it is not conducive to the development of mechanical properties of manufactured sand concrete when it exceeds the reasonable range [23]. The research shows that adding limestone powder can effectively inhibit the alkali-aggregate reaction of concrete [24]. Li et al. [25] pointed out that a proper amount of fines can play a filling role in the concrete system, making the cement stone denser, blocking the capillary diffusion channel, and further reducing the shrinkage of concrete.

At present, many studies have been done on the effect of fines content on the fluidity, mechanical properties, and durability of manufactured sand concrete [26,27,28]. However, there are relatively few studies on the durability and mechanism analysis of high-strength manufactured sand concrete with high fines content. In 2021, Guangdong Province issued the “Guangdong Province Implementation Plan for Promoting the Healthy and Orderly Development of the Sand and Gravel Industry [29]”, which clearly proposed to establish a standard system and accelerate the technological innovation of the sand and gravel industry. In response to this policy to promote the develop the manufactured sand industry, this study takes two types of manufactured sand with different lithologies, i.e., granite manufactured sand and limestone manufactured sand, as the research objects. Their effects on durability properties such as resistance to carbonation, chloride ion, and sulfate corrosion were analyzed, and the mechanisms were analyzed from both macroscopic and microscopic aspects. The work in this study discloses the mechanism how the high fines content affects the durability and provide evidence for the possible relaxation of fines content limit in manufactured sand concrete.

## 2. Raw Materials, Methods and Proportions

### 2.1. Raw Materials

**Figure 1:**
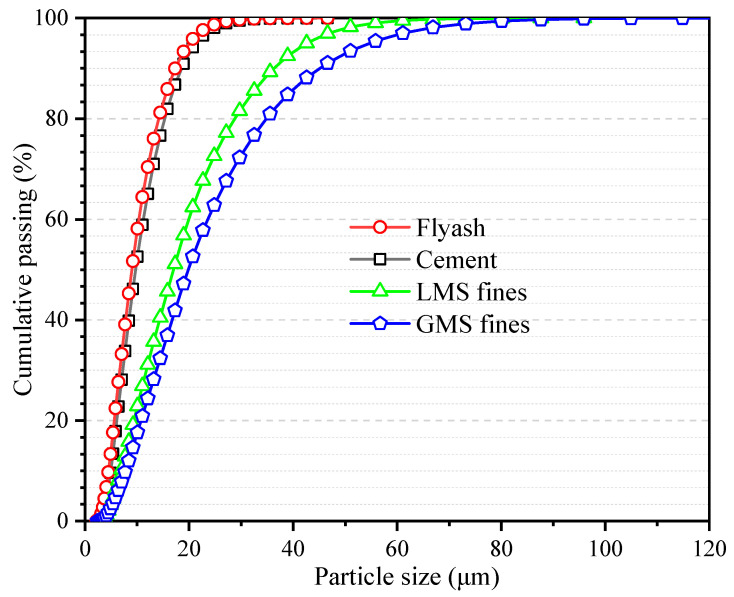
Particle size distribution map of fines, cement, and fly ash.

**Figure 2:**
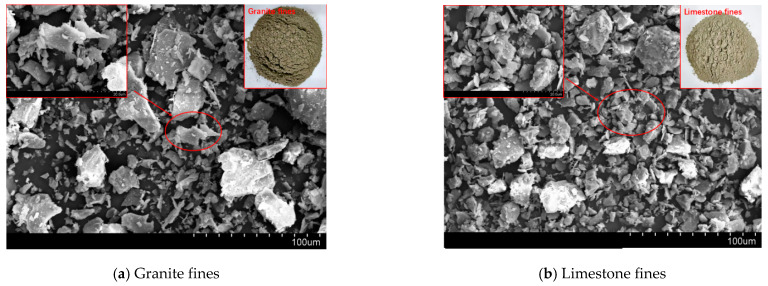
SEM images of the two kinds off fines in this study.

Shijing brand P.O42.5R ordinary Portland cement is used. Type C fly ash from Foshan is used, whose chemical composition is shown in Table 1. Fines is obtained by sieving granite manufactured sand and limestone manufactured sand, and its particle size is lower than 75 μm. The chemical compositions of granite and limestone fines are shown in Table 1, respectively. It can be seen from Figure 1 that the particle size is in the order fly ash < cement < limestone fines < granite fines. Figure 2 shows the microscopic topography of limestone fines and granite fines, both of which are polygonal particles with different shapes.

**Figure 3:**
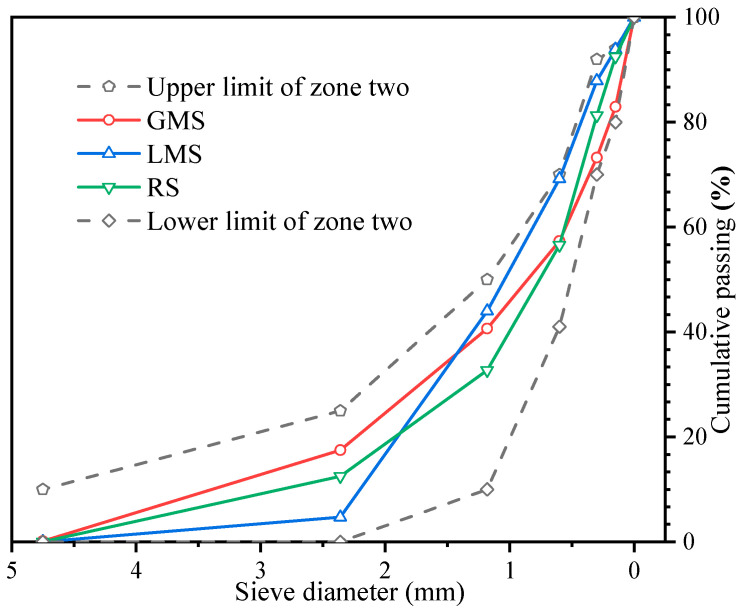
The gradation of sands.

The river sand (RS), granite manufactured sand (GMS), and limestone manufactured sand (LMS) are produced locally in Foshan China. They meet the standard of zone II sand, and their fineness moduli are 2.6, 3.0, and 2.7 respectively. The three gradations of sand are shown in Figure 3. The basic performance indexes of the three kinds of sand are shown in Table 2. The two types of manufactured sand are washed with water to remove fines and dried in an oven. The surface appearance of the three sands and the SEM images at 2000× magnification are shown in Table 3. It can be seen that the surface of the RS is relatively smooth and the cracks are not obvious, while the surface of the two types of manufactured sand is uneven and has obvious cracks. This is because the river sand has experienced long-term water flow erosion and has fewer edges; the manufactured sands have undergone the process of mechanical crushing, and the surfaces have more edges and corners. The surface of GMS is relatively smooth, and the degree of unevenness is relatively gentle; while the surface of LMS is relatively rough, and the degree of unevenness is significant. The coarse aggregate is crushed limestone stone with a particle size of 10–25 mm and 5–10 mm with a mass ratio of 3:7. Admixture using polycarboxylate-based high-performance water reducer. The water is ordinary tap water.

### 2.2. Test Methods

#### 2.2.1. Concrete Carbonation Test

**Figure 4:**
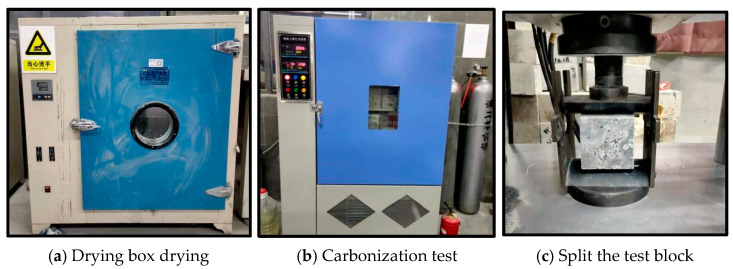

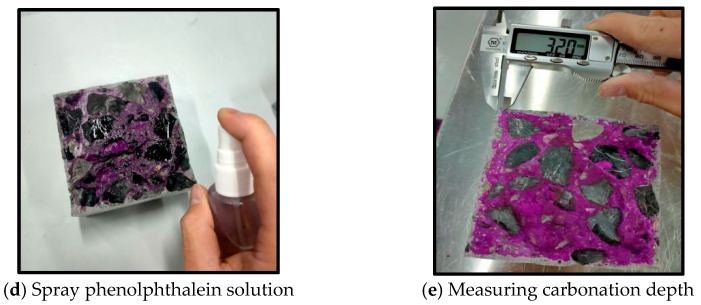
Concrete carbonization.

According to the Chinese standard [30], the carbonation test of C60-manufactured sand concrete was carried out, and a non-standard cubic concrete specimen with a side length of 100 mm was used. The rapid carbonation test of concrete aged 3 d, 7 d, 14 d, and 28 d was carried out. The instrument used in the test is the HTX-12 concrete carbonation box produced by Nanbei Instrument Equipment Co., Ltd. The test process is shown in Figure 4. In the figure, (a) is drying for 48 h; (b) is undergoing carbonization in an oven; (c) is splitting the specimen with furniture; (d) is spraying phenolphthalein solution; (e) is measuring the depth of carbonization.

#### 2.2.2. Anti-Chloride Performance Test

**Figure 5:**
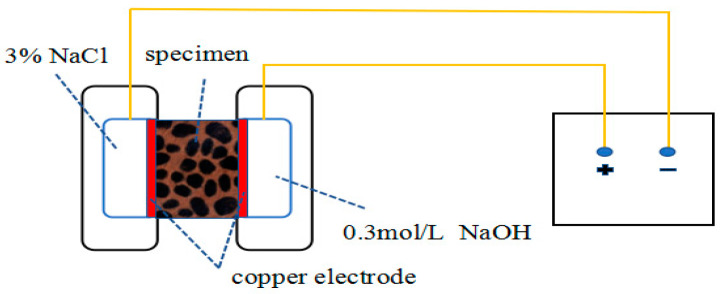
Electric flux schematic diagram.

The electric flux test method is carried out according to Chinese standard GB/T 50082-2009. In this specification, it is recommended that concrete containing a large amount of mineral admixtures can be tested for electric flux at 56 days. Generally speaking, the longer the curing time of concrete, the stronger its permeability resistance. Due to the high content of fly ash in the test ratio, hydration takes a long time to complete. Because the fly ash with a large content in the test ratio takes a long time to hydrate completely, the electric flux tests with ages of 28 days, 56 days, and 90 days are carried out to study the influence of the content of fines on the chloride ion resistance of concrete at the early stage and after hydration. The specimen is a cylinder with a diameter of 100 ± 1 mm and a height of 50 ± 2 mm. Figure 5 is a schematic diagram of the electric flux. Electric flux method test adopted NJ-RCM concrete chloride diffusion coefficient tester from Beijing Durable Weiye Technology Co., Ltd.

#### 2.2.3. Sulfate Long-Term Immersion Test

Sulfate continuous immersion test was carried out on concrete samples with age of 30 d, 60 d, 120 d, and 150 d. The size of the sample is 100 mm × 100 mm × 100 mm cube, 3 pieces for each mix. A 5% Na_2_SO_4_ solution was used in the test. Na_2_SO_4_ was anhydrous sodium sulfate produced by Tianjin Zhiyuan Chemical Reagent Co., Ltd., China. In order to keep the concentration of the solution unchanged, the solution was changed regularly. During the test, the solution was always kept 20 mm higher than the surface of the specimen and the water injection height of the liquid level was kept unchanged.

#### 2.2.4. Compressive Strength Tests

The compressive strengths of concrete are carried out in accordance with the Chinese standard GB/T 50081-2019 “Test Methods for Physical and Mechanical Properties of Concrete” [31].

#### 2.2.5. Packing Density Test

The maximum solid concentration of the solid mix comprised of cement, fly ash, fines, fine aggregate, and coarse aggregate with varying amounts of water is taken as the packing density. In this test, the wet packing method is used to measure the packing density of concrete [32,33].

#### 2.2.6. Mercury Intrusion Test (MIP)

MIP test can analyze the pore structure inside the hardened concrete. The test uses AutoPore IV 9510 automatic mercury porosimeter, the maximum pressure of the mercury porosimeter is set to 413.69 MPa, and the test range of the aperture is 0.003–1000 μm. The fragments with a size of about 10 mm of the broken specimen at 28 d age after standard curing are taken. Then they are dried in a blast drying oven at 40 °C for 24 h before testing.

#### 2.2.7. Scanning Electron Microscope (SEM) Observation

SEM was used to observe the transition zone of concrete interface and the micro-morphology of hydration products. The instrument is an S-4800 electron microscope produced by Hitachi, Japan. The SEM sample is produced as follows: the concrete specimen is cured for 28 days, and the fragments with a size of about 10 mm inside the specimen are taken after the specimen is broken. The fragments are soaked for 24 h to stop hydration, and finally baked in a blast drying oven at 40 °C for 24 h.

#### 2.2.8. Thermogravimetry Analysis (TGA)

TGA is to analyze the changes of substances at different temperatures. According to the weight loss of the substances, it is possible to calculate how much substances are lost. The mass of the test sample is about 20 mg. The temperature rises from 25 °C to 800 °C under the blowing of N_2_ gas, and the heating rate is 10 °C/min. The instrument model is STA 409 PC synchronous thermal analyzer (NETZSCH, Selb, Germany).

### 2.3. Test Proportions

In the test, the water-binder ratio was set constant to 0.3, the total amount of cementitious materials was set constant to 500 kg/m^3^, and the sand to total aggregate ratio was set constant to 44%. According to the actual construction needs, the slump value was set to be 200 ± 20 mm. As can be seen from the water reducer dosage results, a higher fines content results in a lower workability and requires a higher water reducer dosage. With a gradient of 5%, manufactured sands with fines contents of 0%, 5%, 10%, 15%, and 20% were blended to produce concrete samples. A river sand control mix was adopted to study the effect of different fines contents on the performance of C60 manufactured sand concrete after carbonization. The mix proportion is shown in Table 4. Mix GMS-0 means that the GMS is adopted and the fines content is 0%.

## 3. Results and Discussion

### 3.1. Concrete Carbonation Test Results

The carbonation depth results are listed in Table 5. As shown in Table 6, the carbonation depth of all the mixes is 0 at the age of 3 d. When the fines content is 0%, the carbonation depth of river sand concrete at each age (7 d, 14 d, 28 d) is smaller than that of GMS concrete and LMS concrete. The carbonation depth of concrete in the 7 d age mix increased by 136.3% and 218.8% in the GMS and LMS mixes, respectively; for the 14 d age, it increased by 81.1% and 108.8%, respectively; for the 28 d age, it increased by 70.7% and 97.8%, respectively. When the fines content was 5–20%, the carbonization depth of each GMS concrete mix was smaller than that of LMS concrete mix after carbonization for 7 d, 14 d, and 28 d.

The carbonation depth of all the mixes in the test is not large. According to Chinese standard “Concrete Durability Inspection and Evaluation Standard” [34], the standard stipulates that the carbonation depth of concrete in the range of 0.1~10 mm is grade IV. Because the river sand has a good particle size distribution, the river sand concrete has a denser packing effect, and thus has a better anti-carbonation ability. When the fines content is 0%, the GMS and LMS concrete lacks fines to fill the internal pores, and the packing density of the concrete is low and the compactness is poor. This will lead to a larger amount of carbon dioxide entering the interior of the concrete, which will undoubtedly aggravate the carbonization reaction of the concrete. Therefore, the carbonation depth of the two types of manufactured sand concrete without fines is larger.

**Figure 6:**
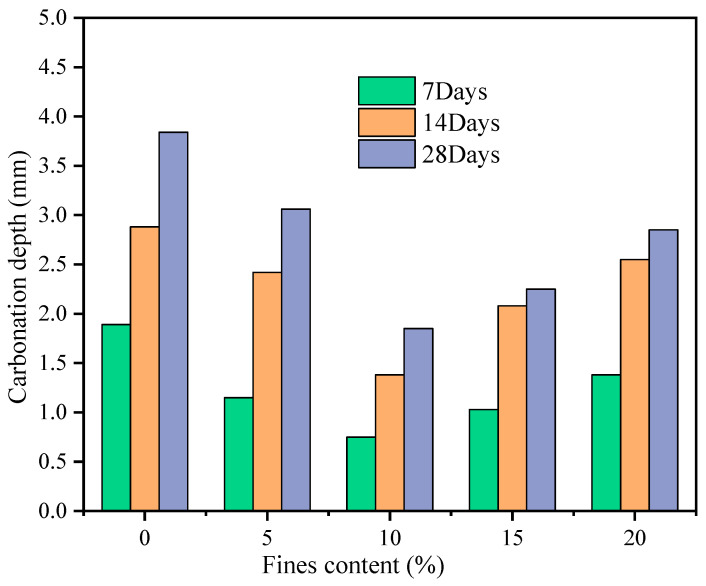
Carbonation depth of GMS concrete with different fines content.

With the change of fines content, the change of carbonization depth of GMS concrete at different carbonization ages is shown in Figure 6. As shown in Figure 6, the carbonation depth of concrete first decreases and then increases as the fines content increases from 0% to 20%. When the fines content is 10%, the carbonation depth of concrete at the age of 7 d, 14 d and 28 d showed the minimum value. Granite fines can fill the pores of concrete in concrete, and the increase of packing density can effectively prevent CO_2_ and H_2_O from entering the concrete, thus improving the carbonization resistance.

**Figure 7:**
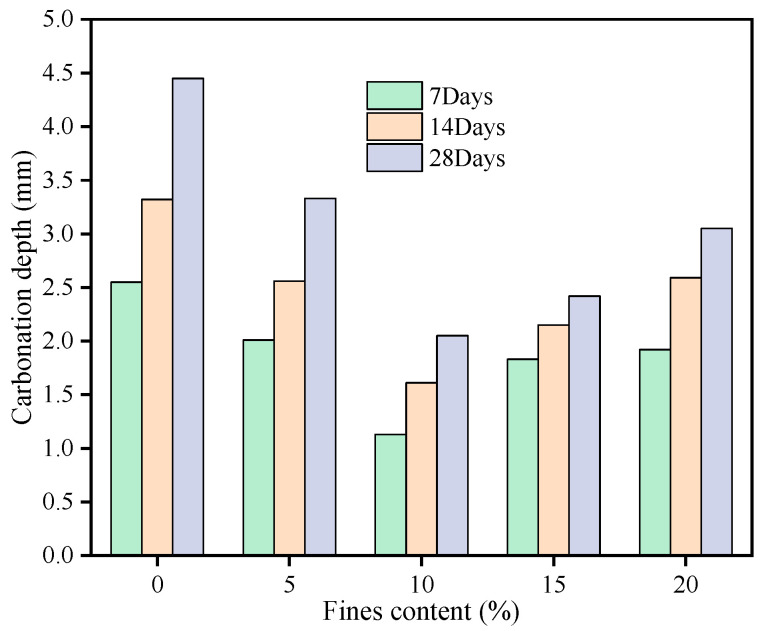
Carbonation depth of LMS concrete with different fines content.

It can be seen from Figure 7 that with the increase in the content of limestone fines, the carbonation depth of LMS concrete at each age (7 d, 14 d and 28 d) also showed a trend of first increasing and then decreasing. When the fines content was 10%, the carbonization depth reached the minimum value. The limestone fines could promote the hydration of cement through nucleation effect. As the hydration product occupied a smaller volume than the total volume of cement and water, the increase in hydration degree would lead to more chemical shrinkage in cement paste. This lowered the volume stability and induced more cracks [35]. In this circumstance, more CO_2_ and H_2_O enter the concrete from the cracks, resulting in deeper carbonization of concrete. Therefore, under the conditions of the same fines content and the same carbonization environment, the carbonation depth of LMS concrete is larger than that of GMS concrete. The limestone fines plays a filling role in the concrete, which lowers the porosity and increases packing density of the concrete, by which the carbonization resistance of the concrete can be enhanced [36]. However, when the fines content is too large, the packing density of concrete will be reduced, which will affect the concrete skeleton structure and lead to an increase in the carbonation depth.

### 3.2. Concrete Anti-Chloride Performance Test Results

As can be seen from Table 6, except for 0% fines content, the electric flux of GMS concrete in other mixes is smaller than that of RS concrete, indicating that when the fines content is 5–20%, GMS concrete has stronger chloride ion penetration resistance than RS concrete. When the fines content is less than or equal to 10%, the electric flux of the LMS concrete for 28 days is higher than that of the RS concrete, and when the fines content reaches more than 10%, the electric flux is smaller than that of the RS concrete. The electric flux value of GMS concrete is lower than that of LMS concrete, indicating that GMS concrete has better resistance to chloride ion than LMS concrete. According to the relevant regulations of the Chinese standard TB 10005-2010 “Code for Durability Design of Railway Concrete Structures”, the electric flux of C60 concrete at 56 d-aged should be less than 800 C, while the maximum electric flux of LMS concrete is 628 C, which meets the specification requirements. This can be partially attributed to the fly ash in the mixes since the alumina and silica in the fly ash can react with the cement to form calcium silicate gel, thereby improving the transition zone of the paste-aggregate interface and consequently the resistance to chloride ion penetration.

**Figure 8:**
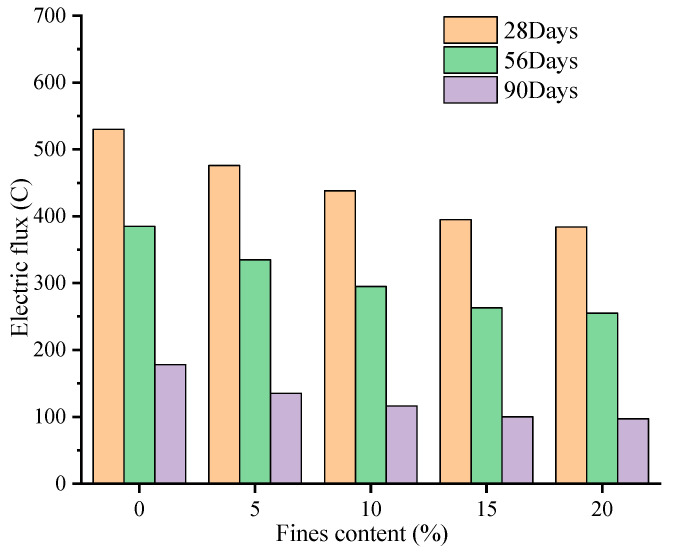
Variation of electric flux of GMS concrete.

**Figure 9:**
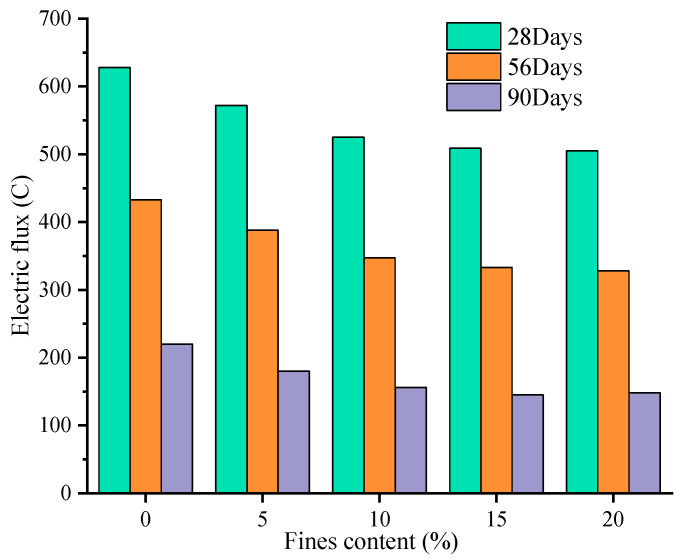
Variation of electric flux of LMS concrete.

**Figure 10:**
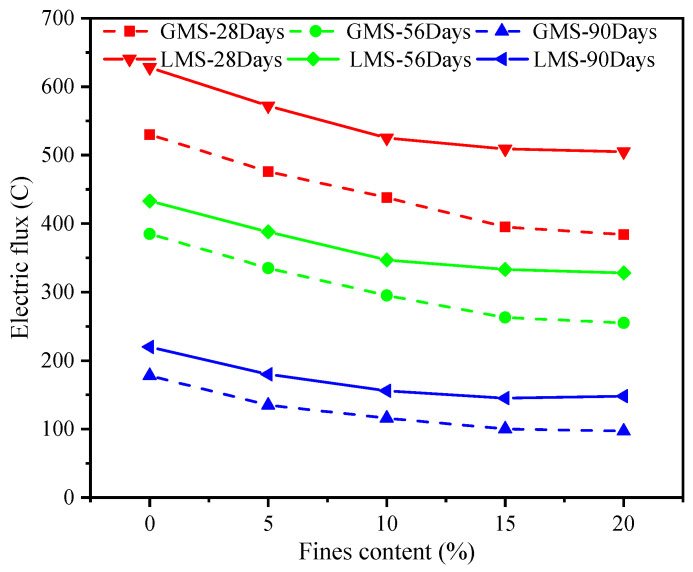
Comparisons of electric flux for GMS and LMS.

Figure 8 and Figure 9 are graphs showing the change of the electric flux value of GMS and LMS concrete with the increase of fines content, respectively. It can be clearly found that with the increase of fines content, the electric flux values of 28 d, 56 d, and 90 d concrete all show a continuous downward trend. It can be seen from the figures that the electric flux of GMS concrete at different ages is smaller than that of LMS concrete. It is worth noting that when the fines content exceeds 15%, the decrease in the electric flux value is very small, indicating that continuous increase in the fines content cannot improve the chloride ion resistance of concrete. Figure 10 shows the comparison of electric flux results of GMS and LMS with the change of fines content at different ages. It can be seen that the electric flux of GMS concrete is lower than that of LMS concrete in the ages of 28 d, 56 d, and 90. It is worth noting that with the increase of age, the difference of electric flux between two kinds of manufactured sand concrete becomes smaller and smaller. The main reason why GMS concrete’s electric flux is lower than LMS concrete is that GMS concrete’s compactness is better than LMS concrete’s, so GMS has stronger chloride ion resistance.

The fact that fines can reduce the electric flux of concrete is also due to the filling effect, which increases the packing density and effectively reduce the amount of chloride ions entering the concrete, thereby improving the resistance to chloride ion penetration of concrete. However, when the fines content is too high, it may adversely affect the resistance to chloride ion of concrete. This is because the volumetric ratio of cement to fines in the concrete decreases, and there are more than enough fines to fill into the voids between cement grain. The packing density is lowered, which results in a decrease in the chloride ion resistance of concrete. Due to the weak conductivity and higher packing density of GMS, the resistance to chloride ion penetration of GMS concrete is higher than that of LMS concrete.

### 3.3. Sulfate Long-Term Immersion Test Results

**Figure 11:**
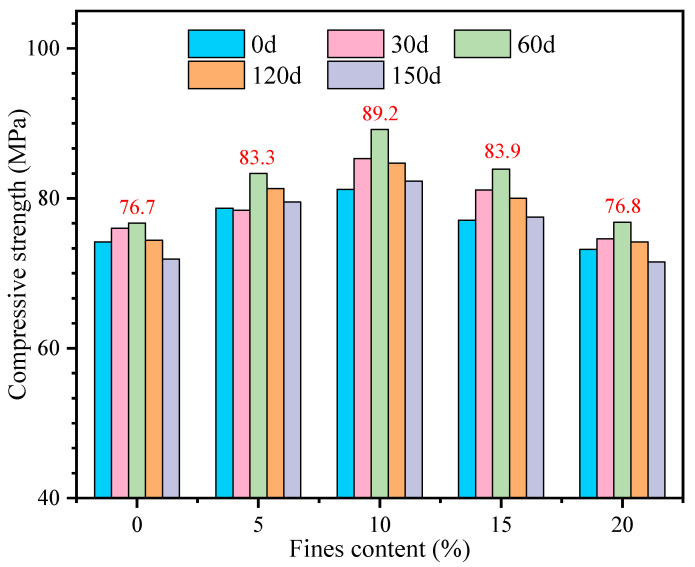
Compressive strength of GMS concrete after continuous immersion.

**Figure 12:**
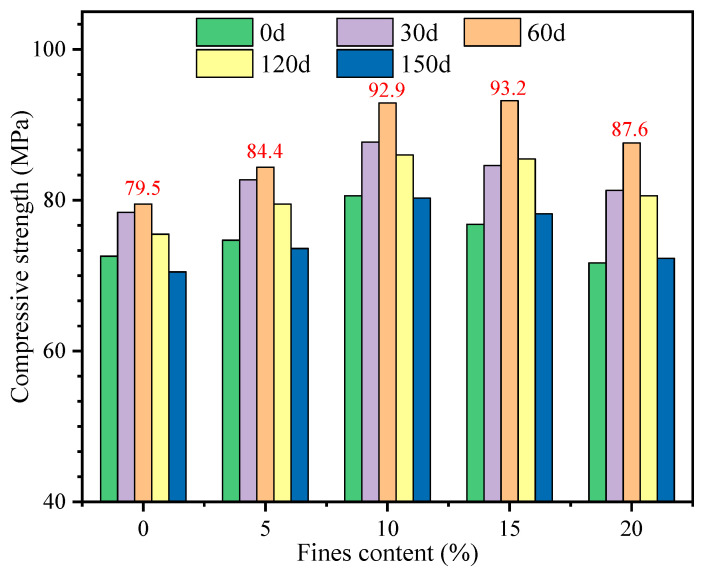
Compressive strength of LMS concrete after continuous immersion.

When the sulfate concentration in the environment where the concrete is located is higher than the solubility of sulfate itself, crystallization will occur. This causes the expansion of concrete several times, and then causes the concrete to crack and break. In this study, a traditional method was used to investigate the effect of different fines contents on high-strength manufactured sand concrete. First, the change of compressive strength under continuous immersion at 30 d, 60 d, 90 d, and 120 d age was studied. The test results are shown in Figure 11 and Figure 12.

It can be found that the compressive strength of manufactured sand concrete with different fines content is improved to a certain extent when immersed continuously for 60 days. This is because the strength development with curing age is more significant than the effect of sulfate attack at early age. However, as the immersion continued, the compressive strength began to decline, that is to say, immersing the manufactured sand concrete for more than 60 days would have an adverse effect on the concrete. When the fines content is 10%, the compressive strength of GMS concrete has a maximum value at each immersion age, and the compressive strength reaches 89.2 MPa when immersed for 60 days. The effects of fines content and immersion age have similar effects on the compressive strength. The highest compressive strength is 93.2 MPa when the fines content is 15% and immersion age is 60 days.

The change of the compressive strength value of concrete after soaking and standard curing at the same age was studied. It can be seen from Table 7 that the compressive strength of GMS concrete under standard curing is higher than that after soaking at the age of 90 d and 180 d. Compared with the standard curing condition, the strength decline of concrete after immersion is as follows: at the age of 90 days, the reduction rate of the compressive strength of the fines content in the range of 5% to 20% is about 4%; and when the age reaches 180 days, the reduction ratio of the concrete compressive strength varies in the range of 6% to 10%. When the fines content is 5~15%, the reduction ratio of compressive strength is stable at around 8%.

It can be seen that the compressive strength of LMS concrete has the similar change as that of GMS concrete at the same age of 90 d and 180 d. The difference is that at the age of 90 days, the reduction ratio of the compressive strength of the LMS concrete with different fines contents is within 2%. At the age of 180 days, the reduction ratio of compressive strength of each mix was more than 17%; the reduction ratios of compressive strength of manufactured sand concrete fines content with 15% and 20% fines content were as high as 24.02% and 20.44%, respectively.

There are two main reasons for the increase of the compressive strength of the two types of manufactured sand concretes with different fines contents after being soaked in sulfate for 60 days. On the one hand, SO_4_^2−^ enters the concrete during the soaking period, and chemically reacts with the cement hydration products to form expansive products such as gypsum and salt crystals, which optimizes the pore structure inside the concrete, thereby improving the compressive strength of the concrete [37]. On the other hand, after 28 days of standard curing, the cement in the concrete has not fully hydrated, and the compressive strength of the concrete will continue to increase with the progress of the hydration reaction. However, as the immersion continues, the expansive products generated inside the concrete continue to increase, and the cracks form and connect with each other, which provides favorable conditions for SO_4_^2−^ to invade the concrete. Therefore, after the immersion to the later stage, the dense structure inside the concrete is damaged and then the compressive strength decreases [38]. An appropriate amount of fines improves the compactness of the concrete, so when the fines content is in the range of 5% to 10%, the compressive strength of the two types of manufactured sand concrete is the highest, and the sulfate corrosion resistance is strong.

## 4. Mechanism Analysis

### 4.1. Packing Density

**Figure 13:**
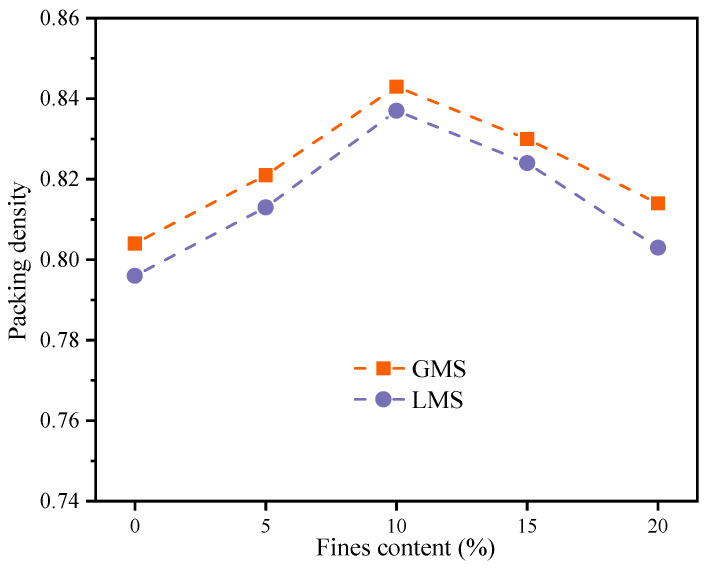
Packing density.

The packing density test was carried out according to the method described above, and the test results are shown in Figure 13. The results show that the packing densities of GMS and LMS concrete both increase first and then decrease with the increase of fines content; when the fines content is in the range of 5% to 15%. And when the fines content is 10%, the packing density of the two types of manufactured sand concrete has the maximum value. It shows that the fines can effectively fill the gaps between the solid materials, and the packing of the concrete is improved. It is verified that a certain amount of fines content can improve the durability of the manufactured sand concrete. When the fines content was higher than 15%, excessive fines for voids filling would decrease the packing density. In addition, when the content of fines is high, the content of free water attached to the surface of solid materials decreases, which may make the slurry unable to effectively fill the pores between aggregates [39], resulting in the decrease of packing density and the decrease of concrete compactness. The changes in packing density with variation of fines content explains the effect on compressive strength.

### 4.2. Concrete Pore Structure

**Figure 14:**
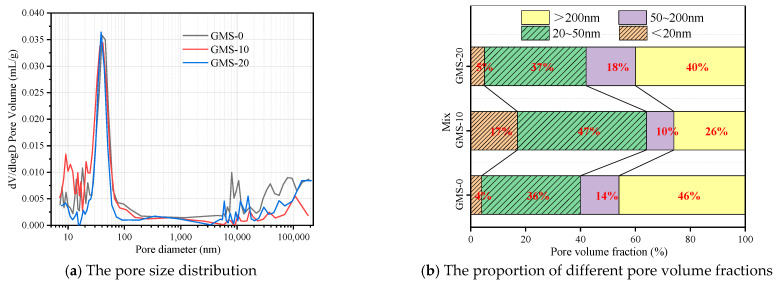
28 d pore size distribution and pore volume ratio of GMS concrete paste.

**Figure 15:**
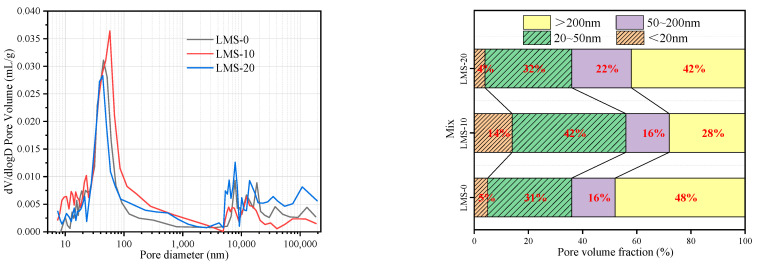
28 d pore size distribution and pore volume ratio of LMS concrete paste.

Three mixes with fines content of 0%, 10%, and 20% were selected for 28 d pure slurry MIP test to further study the effect of fines content on the internal pore structure and compactness of concrete, so as to explain the influence mechanism of fines content on the durability of high-strength manufactured sand concrete. Figure 14 and Figure 15 show the pore size distribution and pore volume ratio of the GMS and LMS concrete pastes, respectively. It can be seen from the pore size distribution map that the pore size distribution of GMS-10 and LMS-10 is mainly in the range ≤ 100 nm. Academician Wu classifies the pore size as follows [40]: the pore size ≤ 20 nm is harmless, and the pore size ≤ 50 nm is less harmful; 50 nm ≤ pore size ≤ 200 nm is harmful hole; the pore size ≥ 200 nm is a harmful pore. With the increase of fines content, the volume ratio of 20~50 nm less harmful pores and <20 nm harmless pores in GMS and LMS concrete first increased and then decreased. When the fines content is 10%, the proportion of harmless and harmless holes in GMS concrete reaches 64%, which increases by 52% and 60% compared with 0% and 20%, respectively. The proportion of harmless and harmless holes in LMS concrete is 56%, which increases by 55% and 60% respectively when the fines content is 0% and 20%. It shows that a certain amount of fines (about 10%) will transform the more harmful and harmful pores in the concrete into harmless and less harmful pores. This also explains the reason why high-strength manufactured sand concrete has strong anti-carbonation, anti-chloride, and anti-sulfate durability when the fines content is 10%.

### 4.3. Aggregate and Cement Interface

**Figure 16:**
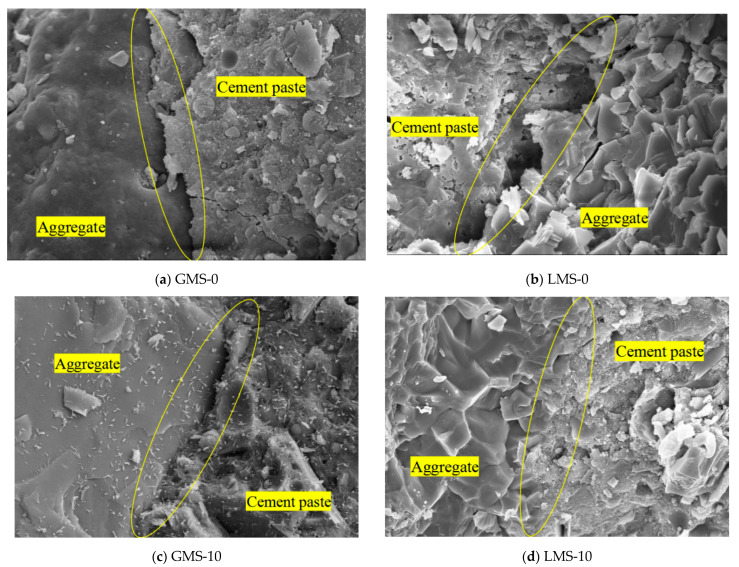

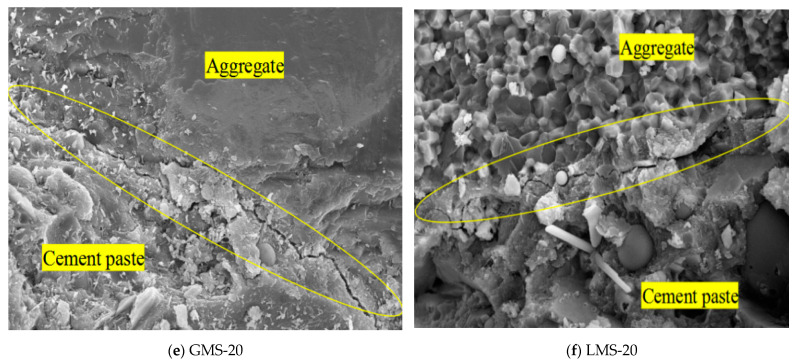
1500 × SEM image of the interface between aggregate and cement.

In order to study the effect of fines content on the interface between aggregate and cement of high-strength manufactured sand concrete, six mixes with fines content of 0%, 10%, and 20% in GMS and LMS concrete were selected for 1500× magnification scanning electron microscope observation. Figure 16 is the SEM image. It can be seen that when the fines content is 0%, obvious cracks can be seen at the interface between the aggregate and cement of the two types of manufactured sand concrete; when the fines content is 10%, the aggregate and cement are well combined and relatively dense, and no cracks are found. When the fines content is 20%, obvious cracks can be seen at the interface between the aggregate and the cement, but it is slightly better than the two mixes with 0% fines content. The research results are consistent with Shen’s results [13]: ITZ of manufactured sand concrete containing a certain amount of fines has almost no obvious cracks, and a proper amount of fines plays a filling role in the concrete, thus reducing the porosity of manufactured sand concrete and improving its performance. The occurrence of a crack when the fines exceed an optimum content is likely due to the lower packing density, which is demonstrated by packing density test shown in Figure 13, and lower amount of free water for hydration of cement and pozzolanic reaction of fly ash when a higher amount of water is absorbed by the fines. When the content of fines is 10%, the good interface between aggregate and cement can improve the durability of the concrete.

### 4.4. TGA

**Figure 17:**
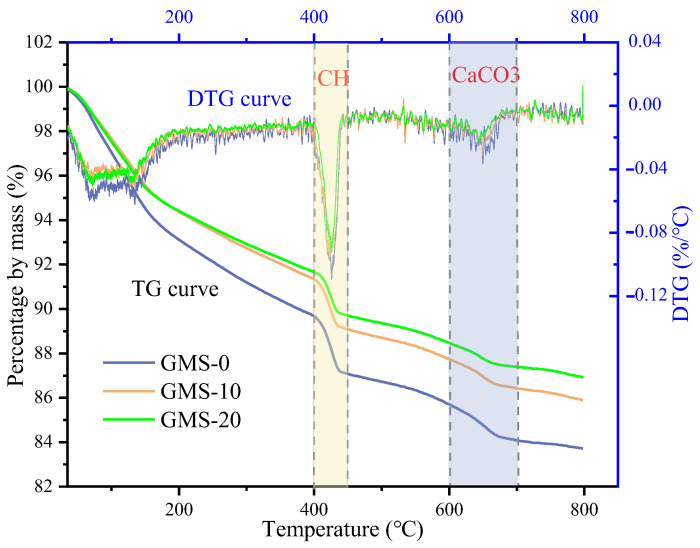
GMS concrete TG and DTG curves.

**Figure 18:**
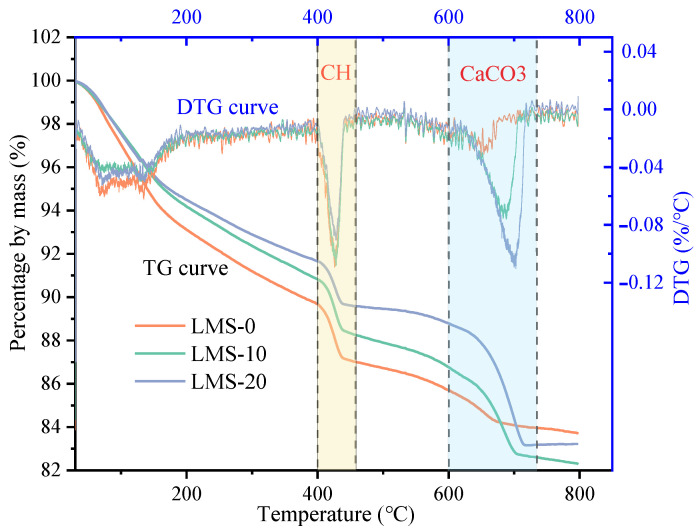
LMS concrete TG and DTG curves.

**Figure 19:**
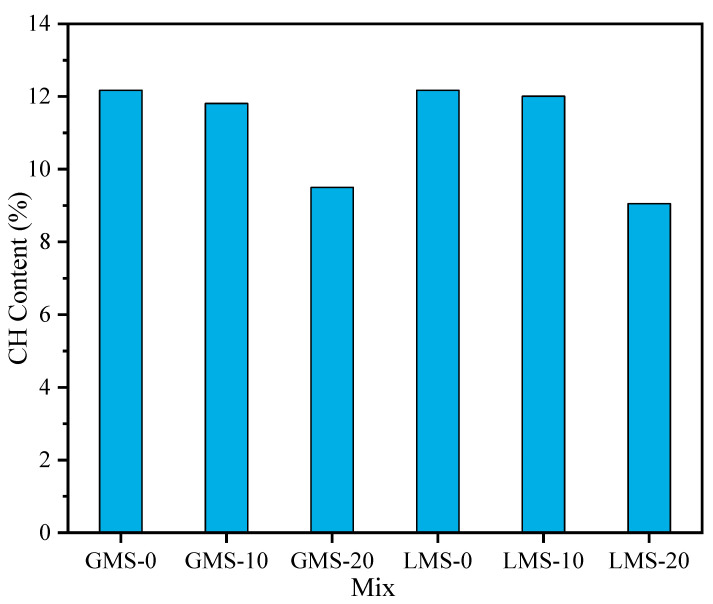
CH content of 28 d pure paste with different fines content.

In order to study the effect of fines content on cement hydration of high-strength manufactured sand concrete, the Ca(OH)_2_ (CH) content was quantitatively analyzed by thermogravimetric method. From Figure 17 and Figure 18, it can be found that different test samples have three obvious weight loss. The weight loss of CH mainly occurs about 400~500 °C. At this time, the CH crystal is decomposed by heat, and the mass of the sample decreases. The mass of CH can be calculated from the lost mass (H_2_O). The calculation result is shown in Figure 19. The results showed that the CH content in the GMS and LMS concrete samples decreased gradually with the increase of the fines content; when the fines content increased from 0% to 10%, the CH content did not decrease significantly, but the fines content increased from 10% to 20%, the CH content of GMS and LMS concrete samples dropped sharply by 19.56% and 24.65%, respectively. This is because a high fines content would absorb a large amount of water, and the lower free water content lead to a lower degree of cement hydration and therefore lower CH content. Granite fines with inert substances such as SiO_2_ and AlO_3_ as the main components will not react with cement and hydration products. However, CaCO_3_ in limestone fines will react with C_3_A in cement [41]. This would lower the CH content generated by cement hydration. Therefore, the CH content in LMS concrete samples is slightly lower than that in GMS concrete samples. The results of this study are consistent with work of Yang et al. [42], who showed that excessive stone powder would inhibit the cement hydration, and the fines mainly plays the role of void filling in concrete. Reasonable utilization of fines can save resources and is conducive to sustainable development.

## 5. Conclusions

This paper studies the influence of fines on the durability of high strength manufactured sand concrete such as carbonation resistance, chloride resistance, and sulfate resistance. The macroscopic test method including packing density tests and the microscopic test method including MIP, SEM, and TGA are used for mechanism analysis. The main conclusions are as follows:

When the fines content is 5~15%, the high strength manufactured sand concrete has good carbonization resistance. When the fines content is 10%, the carbonization depth is the smallest and the carbonization resistance is the strongest. Compared with LMS concrete, GMS concrete has better carbonation resistance.

When the fines content is less than 15%, the electric flux value of the two kinds of manufactured sand concrete decreases with the increase of the fines, and the chloride ion resistance is improved. However, when the fines content exceeds 15%, the chloride ion resistance of manufactured sand concrete will be adversely affected.

The compressive strength of GMS and LMS concrete increases after continuous sulfate soaking for 60 days. After 60 days, the compressive strength decreased gradually with the increase of soaking age. At 90 days, the compressive strength of concrete soaked in sulfate is slightly lower than that under standard curing conditions. When the fines content is larger than 15%, the reduction rate of compressive strength of GMS and LMS concrete is larger than 20% after soaking sulfate and standard curing for 180 days.

The main reason why fines improve the durability of concrete is that the fines plays a filling role in concrete. Appropriate fines content can increase the packing density of concrete, increase the proportion of harmless holes and less harmful holes, and improve the interface bonding between aggregate and cement.

This study demonstrated that an appropriate amount of fines can effectively improve the durability of the manufactured sand concrete, and 10% is the optimal fines content for the highest durability. When the requirements are not high, the fines content can be relaxed to 15%, but it is better not to exceed 15%.

## Figures and Tables

**Table 1:** The chemical compositions of cement, fly ash, granite, and limestone fines.

Composition (%)	CaO	SiO_2_	MgO	Al_2_O_3_	Fe_2_O_3_	K_2_O	LOI	Na_2_O	Else
cement	60.20	24.53	3.32	5.01	3.85	0.28	1.85	0.17	0.79
fly ash	11.52	50.85	1.56	17.21	13.14	1.32	--	0.52	3.88
granite fines	3.58	65.46	2.13	17.82	2.84	2.98	--	3.88	1.07
limestone fines	52.22	3.12	3.01	1.18	1.05	0.41	37.08	--	0.78

**Table 2:** Basic performance indexes of three sands.

Sand Type	Apparent Density kg (/m^3^)	Void Ratio (%)	Fines Content (%)	Methylene Blue Value	Fineness Modulus	Crush Value (%)	Needle-like Content (%)	Water Absorption (%)
RS	2680	34	-	-	2.60	19.2	-	8.56
GMS	2773	38	9.5	0.75	2.72	18.0	3.89	13.02
LMS	2760	39	8.4	2.75	3.00	25.0	4.17	11.75

**Table 3:** Surface morphologies and SEM images of three sands.

Sand Type	Surface Topography and SEM Images
RS	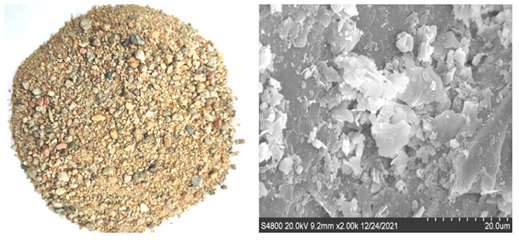
GMS	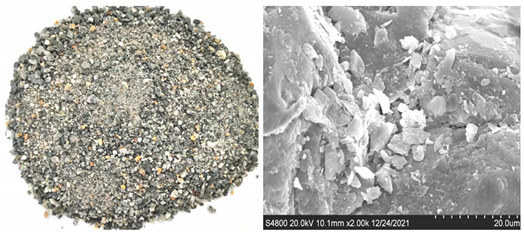
LMS	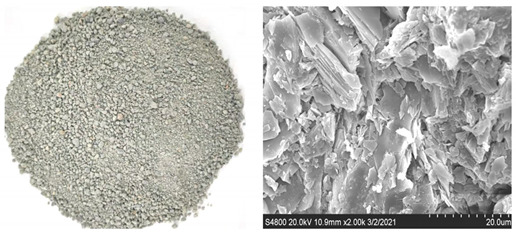

**Table 4:** C60 manufactured sand concrete mix proportion.

Mix	Fines Content (%)	Cement (kg/m^3^)	Fly Ash (kg/m^3^)	Sand (kg/m^3^)	Fines (kg/m^3^)	Gravel (kg/m^3^)	Water (kg/m^3^)	Water Reducer (kg/m^3^)	Slump (mm)
RS-0	0	350	150	805	0	1025	150	3.8	210
GMS-0	0	350	150	805	0	1025	150	2.9	205
GMS-5	5	350	150	765	40	1025	150	3.3	200
GMS-10	10	350	150	725	80	1025	150	4.5	205
GMS-15	15	350	150	684	121	1025	150	5.5	210
GMS-20	20	350	150	644	161	1025	150	6.8	190
LMS-0	0	350	150	805	0	1025	150	3.2	200
LMS-5	5	350	150	765	40	1025	150	3.4	195
LMS-10	10	350	150	725	80	1025	150	3.5	200
LMS-15	15	350	150	684	121	1025	150	4.6	205
LMS-20	20	350	150	644	161	1025	150	5.2	210

**Table 5:** Carbonation test results.

Mix	Fines Content (%)	Concrete Carbonation Depth (mm)			
		3 d	7 d	14 d	28 d
RS-0	0	0	0.80	1.59	2.25
GMS-0	0	0	1.89	2.88	3.84
GMS-5	5	0	1.15	2.42	3.06
GMS-10	10	0	0.75	1.38	1.85
GMS-15	15	0	1.03	2.08	2.25
GMS-20	20	0	1.38	2.55	2.85
LMS-0	0	0	2.55	3.32	4.45
LMS-5	5	0	2.01	2.56	3.33
LMS-10	10	0	1.13	1.61	2.05
LMS-15	15	0	1.83	2.15	2.42
LMS-20	20	0	1.92	2.59	3.05

**Table 6:** Electric flux test results.

Mix	Fines Content (%)	Concrete Electric f€ (C)		
		28 d	56 d	90 d
RS-0	0	511	351	165
GMS-0	0	530	385	178
GMS-5	5	456	315	125
GMS-10	10	438	295	116
GMS-15	15	395	263	100
GMS-20	20	384	255	97
LMS-0	0	628	433	220
LMS-5	5	562	388	170
LMS-10	10	545	377	166
LMS-15	15	509	333	145
LMS-20	20	505	328	141

**Table 7:** Concrete compressive strength concrete at the same age.

Number	Fines Content (%)	Concrete Compression Strength (MPa)			
		Standard 90 d	Standard 30 d + Soak 60 d	Standard 180 d	Standard 30 d + Soak 150 d
RS-0	0	83.9	82.6	86.2	74.2
GMS-0	0	78.3	76.7	84.4	76.9
GMS-5	5	86.9	83.3	88.5	81.9
GMS-10	10	92.7	89.2	95.6	87.8
GMS-15	15	87.3	83.9	91.4	83.7
GMS-20	20	80.4	76.8	87.1	83.7
LMS-0	0	80.6	79.5	87.0	79.8
LMS-5	5	84.9	84.4	90.5	82.2
LMS-10	10	93.8	92.9	97.1	82.0
LMS-15	15	94.8	93.2	102.9	88.3
LMS-20	20	89.5	87.6	90.9	78.7

## Data Availability

No date were used to support the study.
